# Participation in Cancer Clinical Trials: Researching the Causes of Low Accrual

**DOI:** 10.6004/jadpro.2016.7.2.1

**Published:** 2016-03-01

**Authors:** Pamela Hallquist Viale

Clinical trials offer seriously ill patients a chance at receiving investigative therapies containing new or innovative treatments for cancer. These trials can yield extremely valuable information regarding optimal therapies for specific cancers. Yet it’s baffling to consider the fact that less than 5% of adults with cancer participate in clinical trials. And even more baffling is the fact that despite enrollment in clinical trials, almost one in five publicly funded clinical trials in oncology fails to recruit enough participants to provide reliable data.

As oncology advanced practitioners, we sometimes care for patients on clinical trials. Those of us who work in academic or community centers are familiar with the rigors and characteristics of clinical trial participation. Still others of us will help patients obtain referrals to clinical trials offered by other institutions. Caring for patients on clinical trials requires adherence to strict guidelines and reportage.

## ACHIEVING IDEAL ACCRUAL

Unfortunately, securing adequate patient accrual in phase III trials is difficult. A recent study determined that one-third of Clinical Trials Cooperative Group phase III trials closed with insufficient accrual (Schroen et al., 2012). Bennette et al. note that trials with inadequate enrollment numbers represent a waste of resources balanced against little to no contribution to our medical knowledge (Bennette et al., 2015). The researchers studied the empirical relationship and predictive factors related to risk factors representing low accrual in the National Cancer Institute’s Cooperative Group Program (National Clinical Trials Network [NCTN]). The results of the study led to the development of an algorithm to predict factors indicating difficulty in attracting adequate numbers of enrollees for specific clinical trials. The potential risk factors were identified by an expansive literature search.

## STUDY METHODS AND RESULTS

The researchers examined data from 787 phase II/III adult NCTN-sponsored trials that had started between the years 2000 and 2011. The study defined low accrual trials as those that closed with or accrued less than 50% of their target enrollees. The potential predictors were determined from the literature review and interviews with experts; the final predictors were identified using stepwise regression. All statistical tests used for the study were two-sided. Eighteen percent of the NCTN-sponsored trials closed with low accrual (less than 50% of their targets) 3 or more years after initiation of the study. The results demonstrated that a model of 12 trial-level risk factors had calibration for prediction of trials with low accrual and predictor selection strategies (Bennette et al., 2015). Some of the risk factors identified are as follows:

- Trials requiring patients to give a tissue sample or undergo a biopsy
- Trials in which patients know they are unlikely to receive a potentially new drug or therapy (i.e., randomization for investigational treatments)
- Phase III trials (phase II trials are more likely to hit accrual targets than phase III trials)

The researchers note that the algorithm developed from the study can help to predict how an NCTN trial might have better success in enrolling participants and how those factors could be incorporated into trial design.

## IMPLICATIONS FOR ADVANCED PRACTITIONERS

Difficulties in achieving optimal patient accrual numbers for adult oncology clinical trials have been an ongoing problem in cancer care. Much of the research in this area previously focused on the deterrents noted by physicians and patients affecting accrual once the trial has opened (Schroen et al., 2011). The study discussed above represents a new focus on the specifics of trial design at the development phase, illustrating predictive factors that affect accrual numbers prior to the initiation of a clinical trial. The positive effects of higher accrual are many, including a potential for improved results and a more effective way of determining new improvements for cancer care (Schroen et al., 2011). Problems with accrual consist of additional factors not included in the study above; inadequate recruitment resources and high costs to patient and institution have been described previously as barriers to clinical trial entry (Schroen et al., 2011).

Those advanced practitioners working in centers or community practices should be aware of difficulties in patient accrual for selective trials and the need for higher participation to gain adequate information to affect cancer therapy. The algorithm developed by Bennette and colleagues represents an effort to identify predictive factors affecting potential accrual, and incorporation of these factors may aid in optimal trial design. Patients with cancer may have improved outcomes when enrolled in clinical trials. Better design can and should increase those numbers, offering patients improved cancer therapy.
